# Comparison of the immune response induced in mice experimentally sensitized with genetically modified MON810 maize vs its conventional counterpart

**DOI:** 10.1186/2045-7022-1-S1-O21

**Published:** 2011-08-12

**Authors:** Karine Adel-Patient, Valeria Guimaraes, Marie-Françoise Drumare, Sandrine Ah-Leung, Hervé Bernard, Christophe Créminon, Jean-Michel Wal

**Affiliations:** 1 INRA, Unité d'Immuno-Allergie Alimentaire, Gif-sur-Yvette, France; 2 CEA, Service de Pharmacologie et d'Immunoanalyse, Gif-sur-Yvette, France

## Background

The introduction on the market of genetically modified (GM) foods has raised the question of the assessment of the potential allergenicity of the newly expressed protein(s) and of the whole GM food. We aimed at comparing the immune responses induced in mice after experimental sensitization with the insect resistant GM maize MON810 expressing the Cry1Ab protein vs its conventional counterpart.

## Methods

BALB/cJ mice were experimentally sensitized with whole protein extracts from MON810 or non-GM (i.e. Tietar) maize via the intra-gastric (i.g.) or intra-peritoneal (i.p.) routes using Cholera toxin or Incomplete Freund's adjuvant, respectively. Specific humoral immune responses induced were analysed by measuring anti-maize and anti-Cry1Ab antibody productions using specific immunoassays and western blotting. Cellular response was assessed by quantification of the cytokines secreted after *ex vivo* reactivation of splenocytes from sensitized mice using protein extracts from GM or non-GM maize and purified Cry1Ab.

## Results

Efficient sensitization was achieved in mice administered maize protein extracts. Humoral and cellular immune responses against endogenous maize proteins were quantitatively equivalent in mice treated with MON810 vs its non GM counterpart. No anti-Cry1Ab immune response was detected in mice that received MON810 maize. Although the pattern of recognition of maize proteins by IgG antibodies differed in i.p. vs i.g. sensitized mice, no difference was evidenced between treatment by MON810 or its non-GM comparator when considering the same sensitization route.

## Conclusion

No significant unintended effect of the genetic modification has been evidenced on the immune responses induced in mice after experimental sensitization by MON810 maize using the i.p. or i.g. route.

